# Improving the provision of services to young people from refugee backgrounds with comorbid mental health and substance use problems: addressing the barriers

**DOI:** 10.1186/s12889-017-4186-y

**Published:** 2017-03-24

**Authors:** Miriam Posselt, Karalyn McDonald, Nicholas Procter, Charlotte de Crespigny, Cherrie Galletly

**Affiliations:** 1 Discipline of Psychiatry, School of Medicine, University of Adelaide, Level 4, Eleanor Harrald Building, Frome Rd, Adelaide, South Australia Australia; 2 Jean Hailes Research Unit, School of Public Health and Preventive Medicine, Monash University, Melbourne, VIC Australia; 3 School of Nursing and Midwifery, University of South Australia, Adelaide, South Australia Australia; 4 School of Nursing, University of Adelaide, Adelaide, South Australia Australia; 5 Ramsay Health Care, Adelaide, Australia; 6 Northern Adelaide Local Health Network, Adelaide, Australia

**Keywords:** Refugee, Youth, Comorbidity, Service provision, Mental health, Substance use

## Abstract

**Background:**

South Australia (SA) has resettled 151,134 refugees in the last ten years (Department of Immigration and Border Protection, Settlement reporting facility, 2014). Northern metropolitan Adelaide, an area which experiences significant social disadvantage, has received a significant number of (predominantly young) refugees. Research indicates that refugee youth are at elevated risk of mental health (MH) and alcohol and other drug (AOD) problems. These factors, along with the low socio-economic status of northern Adelaide, the number of refugee youth residing there, and the added complexity of treating comorbid MH and AOD problems (comorbidity) prompted this research. We investigated the barriers and facilitators to culturally responsive comorbidity care for these youth and whether the MH and AOD services were equipped to provide such support.

**Methods:**

This mixed-methods study employed semi-structured interviews with refugee youth and service providers and an online survey with managers of services. Thirty participants (15 refugee youth, 15 service providers) took part in the semi-structured interviews and 56 (40 complete, 16 partially-complete) in the survey.

**Results:**

Thematic analysis of the interview data revealed the most commonly reported barriers related to four broad areas: (1) organisational and structural, (2) access and engagement, (3) treatment and service delivery, and (4) training and resources. Survey data supported the barriers identified in the qualitative findings.

**Conclusions:**

This research highlights significant gaps in the response of MH and AOD services to refugee youth with comorbidity. Based on the findings, ways of overcoming the barriers are discussed, and are of particular relevance to policy makers, organisations and clinicians.

## Background

Comorbidity is defined as the existence of one or more clinical conditions [1]. The use of the term “comorbidity” in this research refers exclusively to the co-existence of mental health (MH) and alcohol and other drug (AOD) problems (also commonly referred to as dual diagnosis). Comorbidity is prevalent among the general population of Australia and treatment for individuals with comorbidity is often complicated by challenges relating to detection, diagnosis and treatment, including the separation of MH and AOD service sectors [1, 2]. While we have a growing understanding of the implications of these challenges for treatment of individuals in the general population, there is little knowledge concerning how these issues impact on individuals from refugee backgrounds.

### The significance of comorbidity

The aetiology of comorbidity is complex. Research suggests there are three main explanations as to how comorbidity occurs; that causal relationships are either direct or indirect or that common reasons lead to both conditions developing [1, 3]. Establishing the cause of comorbidity and determining which disorder came first may be useful in understanding the development of the problem, which can therefore be addressed during treatment [1]. However, regardless of causal relationship, it is understood that each condition assists in maintaining or exacerbating the other and that addressing both conditions is critical [4]. Research has consistently reported that individuals with comorbidity experience poorer prognoses, premature mortality, higher rates of suicide, a more severe illness course, greater burden of disability, difficulty obtaining correct diagnoses, greater difficultly accessing effective treatments and greater use of health services than those with only one disorder [4–7]. Many reports and guidelines have been produced in an attempt to improve the outcomes for individuals with comorbidity. The majority of the literature states that integrated and coordinated treatment models addressing both conditions (usually concurrently) are necessary, see, for example, Allsop [1], Donald et al. [8], de Crespigny & Talmet [9], and Gordon [10].

### The prevalence of comorbidity

Large-scale prevalence studies report high rates of comorbidity. The National Comorbidity Survey in the United States found that in a sample of 8100 people, 41–65% of people with an addictive disorder had at least one mental disorder and 51% of those with a mental disorder had at least one addictive disorder [11]. In Australia, MH problems are prevalent among clients of AOD services and AOD use is more common among those with MH diagnoses than in the general community [1, 2]. The 2013 National Drug Strategy Survey found that the prevalence of mental illness was greater among adults who had used illicit drugs within the past 12 months (21%) or past month (24%) than those who had not used (12.6%) [12]. Comorbidity is also an important issue among young people in Australia. According to the most recent Mental Health and Wellbeing Survey (2007), 26% of young people (aged 16–24 years) had a recent (within the past 12 months) mental disorder and 13% reported a recent substance use disorder [13]. Young people with a recent mental disorder (36%) were five times more likely than those without mental disorders (7%) to have misused drugs in the previous year. Further, large proportions of those with mood (37%) and anxiety (32%) disorders reported misusing drugs within the last 12 months. The most common substance use disorder found among young people was harmful use of alcohol (9%) and 57% of those with a recent mental disorder reported consuming alcohol at least weekly compared to 35% of those without a mental disorder. It is difficult to determine the extent to which these findings are generalisable to young refugees in Australia.

Refugee young people face multiple risk factors before, during and post-migration, placing them at risk of MH and AOD disorders [14–19]. There are high rates of posttraumatic stress disorder (PTSD), depression and other psychiatric problems among refugee groups. Prevalence studies concerning refugee young people have reported that rates of PTSD vary from 19 to 54% and rates of depression vary from 3 to 30% [20]. There are well-established links between PTSD and AOD disorders [21], as well as between socio-economic disadvantage and AOD use among migrant populations [22, 23].

### Service utilisation

Many individuals with comorbidity do not access treatment or support [24]. This is reported to be the case for young people in Australia [13, 25] and for culturally and linguistically diverse (CALD) individuals across the lifespan [26]. In Australia, only 23% of young people in the general community who reported a MH problem in the previous 12 months had accessed a service within that time period and young people with AODs were even less likely to have accessed formal support (11%) [13]. Young refugees in particular are underrepresented in support services and face substantial barriers accessing support and treatment for both MH [27–29] and AOD problems [30–32]. Given these findings, it is likely that young people from refugee backgrounds with comorbidity face additional obstacles to appropriate assessment, support and treatment. Research conducted in Sydney, Australia investigated the barriers to accessing services by culturally and linguistically diverse people with comorbidity [33, 34]. Flaherty and colleagues (2012) interviewed service providers and clients and found that services not only struggle to effectively help those with comorbid MH and AOD conditions but also fail to adequately accommodate cultural and linguistic diversity [33]. There has been no such research looking specifically at young people from refugee backgrounds. However, it is likely that the complications concerning ethnicity and cultural diversity would apply to refugee youth. Further, these difficulties may be compounded by the backgrounds of these young people as many will have had traumatic experiences, by the very definition of refugee [35]. This paper goes some way towards addressing the paucity of research concerning comorbidity among refugee youth.

### The study region

The local government areas (LGAs) of Salisbury and Playford in the northern suburbs of Adelaide experience significant social disadvantage. At the time this research was conducted, the Australian Census of Population and Housing (2011) reported high unemployment rates in Salisbury (6.97%) and Playford (8.01%) compared to the rest of South Australia (5.31%), and Australia (5.65%) [36]. In June 2013 greater proportions of families residing in both Salisbury (14.8%) and Playford (23.6%) were assessed as low income and welfare dependent compared with the SA (10.1%) or Australian (9.8%) average [37].

Over the last decade SA has resettled 151,134 refugees under the humanitarian program [38]. One third of these arrivals were resettled in the LGAs of Salisbury and Playford. Almost two thirds (63%) of these refugees were under the age of 25 years on arrival. To our knowledge, there has been no investigation into whether the MH and AOD services are equipped to respond appropriately to clients from refugee backgrounds.

### The present study

This research aimed to determine the barriers and facilitators to effective, culturally responsive service provision for young people of refugee background living in the study region with comorbid MH and AOD problems. The findings from this research can be utilised to make changes in policy and practice to overcome these barriers.

## Method

This mixed methods study involved three components of data collection:

1. Interviews with consumers (refugee youth)
2. Interviews with service providers (‘on the ground’)
3. Online survey of MH, AOD and related services (management staff)

### Mixed methods

This research employed a sequential exploratory mixed methods design where the qualitative component informed the development, analysis and interpretation of the quantitative survey. The survey aimed to provide additional data to the interview findings, and to help verify the qualitative findings. The use of more than one approach to investigate a research question, known as data triangulation, was important because it enabled comparison of findings from the different data sources and methods and ensures greater confidence in the findings. A mixed methods approach to address a complex area provides valuable data because it draws on the strengths of both methods and allows us to compare findings from different perspectives [39].

### Participatory action research

This research drew on principles of participatory action research (PAR) by involving community members and participants in all stages of the research process [40]. Numerous meetings were held with community members, stakeholders and refugee advocates to optimise the research objectives, the interview guides, provide valuable assistance with recruitment, and provide insight into the findings. This approach facilitated the recruitment and data collection process and provided the community with an opportunity to contribute to the research and have their perspectives heard and considered at every stage of the research. This process also assisted with the interpretation and validation of the results through the sharing and discussion of findings with the members of the community via a group discussion (*n* = 3), individual consultation (*n* = 2) and a meeting of local health professionals (*n* = 10; 2 of whom were participants). Although a PAR study would typically involve an intervention or ‘action’ phase of the research after having engaged with the community, collected data, and shared with findings back to the community, intervention was beyond the scope of this study. However, as this research was situated within a larger project, our specific findings then informed the delivery of workshops for health professionals regarding local service provision for individuals with comorbidity [41].

Ethics approvals were obtained from the Women’s and Children’s Health Network Human Research Ethics Committee and The University of Adelaide Human Research Ethics Committee. All interview participants or their guardians gave written informed consent and survey participants indicated their consent online before continuing to the questions.

### Qualitative component

#### Sampling and recruitment

The relationships established using the PAR approach with community leaders, members and advocates facilitated recruitment of both refugee background young people and service providers. The principal researcher (MP) was invited to attend various community events to hand out flyers and promote the study to health professionals and to people from refugee communities. Interview participants were recruited using purposive sampling by identifying individuals who possessed knowledge related to the research question and who would be able to provide rich, in depth information. Using snowball-sampling techniques, participants were asked to identify other relevant individuals who could potentially participate. Some refugee youth participants (*n* = 4) were encouraged by their MH or AOD workers to participate in the study as they were identified as being able to advocate for refugee background youth with comorbidity. In addition to using the established networks to recruit service providers, MH, AOD and refugee support services were contacted and professionals were invited to participate.

#### Data collection

Interviews with service providers and refugee youth were conducted during 2013 and 2014. Interviews were conducted in locations where the participant felt most comfortable including libraries, cafes, various health services and the local migrant resource centre. Interviews were semi-structured and an interview schedule was used. The interviewer used prompts, probes, clarification, and follow-up questions to enable deeper exploration of the participants’ knowledge and lived experiences. Questions were broadly focused on the difficulties for refugee youth resettling in Australia, specific risk factors related to the development of MH and AOD problems and comorbidity, challenges youth face once they are experiencing MH and AOD problems and comorbidity, and barriers and facilitators perceived to impact on access to services and treatment for youth with comorbidity. Interviews ranged from 45 to 90 min in length, were audio-recorded and transcribed verbatim. All interviews were conducted and the majority were transcribed by the principal researcher to allow total immersion in the data. Refugee youth who participated in the interview received a AUD$20 shopping voucher to compensate for the time spent participating and travel costs.

#### Analysis

Data were analysed using a thematic approach guided by a commonly applied protocol for thematic analysis [42] with the assistance of NVivo 9 software [43]. Data were initially coded and then re-coded as additional themes emerged. A coding structure was determined where coded categories were collapsed and organised with notes identifying their relationships to other codes and overarching themes. All emergent themes (derived from the corresponding codes) were then categorised under the broad over-arching themes presented in this article.

#### Validity checking of the qualitative data

In addition to utilising triangulation techniques, employing a mixed methods design, and consulting the community to verify the findings, potential biases of the principal researcher were addressed by regular meetings with the authors to discuss and interpret the qualitative findings. Individual transcripts and emerging themes were discussed to develop and enrich the interpretations and subsequent conclusions.

### Quantitative component

#### Recruitment

An initial scoping study was conducted within the larger parent project and is published elsewhere [44]. This scoping study identified 70 services which provided treatment and support for individuals with MH or AOD problems living in northern Adelaide. Of these, 26 services were relevant for young people from refugee backgrounds. These services were contacted and email addresses were obtained. An email was then sent with study information and a link to the survey. Workers employed in a management or leadership role at a MH, AOD or related service, which provided support or treatment for youth aged 12–25 years in northern Adelaide, were eligible to participate in the 10–20 min online survey. By way of snowball sampling, participants were encouraged to forward the link and email to other eligible colleagues. Given that participation in the survey was anonymous and that there may be a number of management positions within each service, we are unable to estimate how many services took part in the survey. However, this survey did not aim to generate a representative sample but rather, aimed to collect information from a number of managers from various services. Therefore this recruitment technique was adequate for the purposes of our exploratory research.

#### Data collection

The survey consisted of 35 questions regarding service provision for refugee background clients aged 12–25 years. The questions concerned staff training, data collection and access to resources, funding and interpreters as well as asking participants to identify barriers and facilitators within their organisation to culturally responsive care for this population. The survey was conducted from May to July 2014.

#### Data analysis

Data were analysed using SPSS [45]. The analyses were guided by findings from the qualitative data. For example, interview participants reported marked differences in the way refugee youth were treated, as well as the cultural competence of the staff, depending on the type of MH or AOD organisation. Further analyses examined differences in responses between Government Organisations (GOs) and Non-Government Organisations (NGOs) using Chi-square tests. Where the assumptions of the Chi-square test were violated (such as if the cell count was less than five) Fisher’s exact test of significance was calculated to determine if there were significant differences between groups. A *p* value less than .05 was considered significant. Cohen’s [46] definition of effect size was used, with 0.2 indicating a small effect, 0.5 a moderate effect and 0.8 a large effect.

## Results

### Participant description

#### Qualitative component

##### Refugee youth

Fifteen people aged between 12 and 25 years (average 17.7 years), 9 female and 6 male, participated. They were from Afghan (60%), African (27% [Congolese, Liberian, Burundian]) and Bhutanese (13%) backgrounds, and had been living in Australia for an average of 4.9 years. Two had arrived as unaccompanied minors.

##### Service providers

Service providers interviewed were from government (*n =* 7) and non-government (*n =* 8) MH, AOD and refugee support services. They were qualified social workers (*n =* 10, 5 of whom were program managers), psychologists (*n =* 2) and mental health nurses/nurse practitioners (*n =* 3).

### Quantitative component

Fifty-six participants took part in the survey (40 complete and 16 partially complete) (Table 1). All were employed in management or leadership positions: team leaders (58.5%, *n =* 24), service managers (26.8%, *n =* 11), section managers (9.8%, *n =* 4) and program managers (4.9%, *n =* 2).

**Table 1:** Service/ participant characteristics

	Government	Non-Government	Total
MH	26	10	36
AOD	1	3	4
Combined MH/AOD	4	5	9
Other (correctional, homelessness, gambling)	3	4	7
Total	34	22	56

*MH* mental health service. *AOD* alcohol and other drug service

### Results from thematic analysis

Four broad themes relating to the barriers and facilitators to effective service provision for refugee youth with comorbid MH and AOD problems were identified:

1. Organisational and structural barriers
2. Access and engagement
3. Treatment and service delivery
4. Training and resources

Within each of these broad themes, a number of subthemes that are described. Many themes and subthemes are interrelated and have been organised in a way which best reflects the reported importance of each of the barriers. There was overall consistency in the perspectives of both groups of interview participants and they are therefore organised and presented together. Any differences or contrasting perspectives are highlighted. Some subthemes were predominantly reported by service provider participants. Where this occurred, the quotes presented are exclusively those of service providers. More commonly, participant quotes are presented from both groups of participants and are identified by either ‘RY’ (refugee youth participant) or ‘SP’ (service provider participant).

### Results from quantitative analysis

Overall, there was convergence between the results of the qualitative and quantitative data. The findings from the total dataset are presented in Table 2 and show the general trends reported by survey participants. However, survey data relating specifically to the qualitative themes are presented within each corresponding or relevant theme to complement and strengthen the research findings.

**Table 2:** Cultural responsiveness of MH and AOD services: Summary of all respondents

	Yes *n* (%)	No *n* (%)	Other (response specified) *n* (%)	Total *n* (%)
Does your service allow home visits?	40 (71.43%)	16 (28.6%)		56 (100%)
Do your clients have access to accredited interpreters?	52 (92.9%)	3 (5.4%)	1 (1.7%) (Unsure)	56 (100%)
Is your service adequately funded to provide treatment to refugee background clients?	17 (30.4%)	39 (69.6%)		56 (100%)
Is your service adequately resourced to provide treatment to refugee background clients?	19 (33.9%)	37 (66.1%)		56 (100%)
Does your organisation collect data regarding if client is of refugee background?	13 (32.5%)	27 (67.5%) [23 (57.5%) Only collect country of birth]		40 (100%)
Do your staff receive any training for working with CALD clients?	26 (65%)	14 (35%)		40 (100%)
Do your staff receive any training for working with refugee background clients?	10 (25%)	30 (75%)		40 (100%)
Does your service employ 1 or more CALD/ cultural liaison/consultation or bi-cultural workers designated to work with CALD clients?	19 (47.5%)	21 (52.5%)		40 (100%)
In your opinion are the staff in your service adequately trained to provide treatment for refugee background clients?	6 (15%)	18 (45%)	16 (40%) (There is room for improvement.)	40 (100%)
Do refugee clients and potential clients experience any barriers to accessing your treatment service?	27 (67.5%)	13 (32.5%)		40 (100%)
Do you think young people from a refugee background have the same access to services as other young clients?	9 (22.5%)	31 (77.5%)		40 (100%)
Do you think young people from a refugee background with drug dependency issues and mental illness get the same level of treatment as people who only experience one or the other?	13 (32.5%)	27 (67.5%)		40 (100%)
Do you think young people from a refugee background with drug dependency issues and mental illness get the same level of treatment as young people in the general population with drug dependency issues and mental illness?	12 (30%)	28 (70%)		40 (100%)

### Theme 1: Organisational and structural barriers

One of the most commonly reported barriers for clients accessing and receiving appropriate comorbidity care was the fragmented structure of services. This fragmentation related to the divide of MH and AOD services, as well as that between mainstream services and CALD/ refugee specific services. Fragmentation increased the experience of ‘run around’ for refugee youth clients, where young people were required to attend multiple services, potentially risking further disengagement. This was highlighted by service providers:

*“Probably one of the biggest problems is the way in which services are funded to work in silos, there is that disconnection.” SP201*

The interviews with refugee youth revealed that they often were unaware of services available to them. Many refugee youth participants indicated they were willing to seek assistance from agencies if they considered that their services would be of benefit:

*“If the services are well known or better known in the migration agencies this could increase access. So if services worked with settlement support agencies they would know where people can get help and give advice.” RY110*

Related to the fragmentation of services, interview participants reported there was an unaddressed need for stronger partnerships and collaborative interagency projects. It was generally agreed that the need for partnerships or collaboration was greater when dealing with refugee communities because some agencies had skills, resources and connections with communities. However, they did not have the ability to take on more clients. In contrast, others had the capacity and desire to take on the clients but faced significant barriers to engaging refugee clients. The lack of collaboration between MH and AOD sectors resulted reduced opportunities for access:

*“I think services need to work in partnership with one another; it needs to be a joint initiative.” SP201*

Service providers often spoke about the need for partnerships and collaboration between organisations as a way of addressing the lack of funding for community engagement:

*“They need to create partnerships with services that do [have funding for CALD community engagement] and can because they are out there. They need to reach out and they also need to be receptive…” SP201*

A further barrier identified by service providers was the lack of funding for improving the cultural responsiveness of their service. The need for these efforts was also recognised by refugee youth. Young people reported that more activities to promote mental health awareness and knowledge of services would reduce stigma and improve access to services. Service providers acknowledged that although they knew these efforts were necessary, limited funding and resources prevented this from occurring:

*“I think one of the things in our current health environment that we are struggling to hang on to, is community engagement.” SP 202*

This finding was corroborated by survey participants who said they believed their service was inadequately funded to provide treatment to young refugee background clients (69.6%, *n =* 39) and inadequately resourced (with bi-lingual materials, assessment tools and so on) to provide treatment for these clients (66.1%, *n =* 37).

The final barrier identified by service providers was the lack of data collection concerning refugee background clients. Most reported that their organisations did not collect any data regarding the background of their clients. They understood this to be because few young refugee clients accessed their services (unless they were specifically funded for refugee clients), and there was no perceived need to collect such data. However, most felt that the collection of this data was important:

*“It [refugee background] is often recorded in consultation but not on the data system and I think that’s a problem across the state.” SP202*

Consistent with reports from interview participants, only a small number of survey participants (32.5%, *n* = 13) reported that their organisation collected data concerning refugee background. However, just over half (57.5%, *n =* 23) of participants reported their service did collect data on country of birth.

### Theme 2: Access and engagement

The second theme from the interview data was access and engagement with services. Given that MH stigma is a problem in the general population and reported to be greater in CALD communities, it was not surprising that shame and stigma associated with experiencing MH and AOD problems was a frequently reported barrier to accessing services. Service providers discussed the stigma associated with mental health issues within the wider community as well as concerns by young people themselves accessing services that have Mental Health in the name of the organisation:

*“I don’t think that this client group readily access mental health services anyway and when you’ve got mental health in your name it’s a real issue.” SP 202*

Young people also spoke about their concerns about being seen by their community members in a service that provides mental health services and the subsequent conclusions that would be drawn by others:

*“If I go alone to hospital and someone saw me and they would say- you have been there, I saw you. You have been attending appointment.” RY106*

Refugee youth reported they had very little knowledge of mental illness, addiction and the potentially harmful consequences of drug and alcohol use, as well as very little awareness of MH and AOD support services. Lack of information, compounded with the fear and mistrust of services, was reported to result in lack of help-seeking:

*“No I couldn’t find anyone, I couldn’t trust anyone…I was embarrassed too but it’s just that there was no one to trust.” RY 114*

There was also agreement among refugee youth that when this information was presented in schools or at community events, it was rarely delivered in a way that was meaningful to CALD or refugee background individuals:

*“…even if you do [receive drug and alcohol education] you might see that as just a Western thing”. RY 115*

Participants suggested an information exchange between service providers and resettled refugee communities as a way of meaningfully engaging refugee background youth in health and support service promotion:

*“If they [MH workers] give lots of information to the refugees, [they] get lots of information from them [refugees] - what kind of situation the refugee people got”. RY106*

Increasing the profile of services, including their work in MH and AOD issues, was considered important by refugee youth as this would not only increase information and education but also foster trust and familiarity:

*“So when they [workers from local MH service] came to the school they introduced themselves, they talk about themselves and that’s when you find them more interesting and can go and see them once you know about them” RY 110*

There was agreement among all participants that fear and distrust was a major barrier to service engagement. This included fear of disclosing personal information, fear of retribution, such as being deported or put back in immigration detention, fear of people in authority related to their previous experiences with corrupt or violent government officials, fear of gossip by interpreters (and reported experiences of this occurring), and fear of clinicians informing parents of their difficulties:

*“Lack of trust also is a big problem and the larger CALD communities, they think if I tell them this will they tell the police? Will they tell the child protection agencies?” SP 206*

Similarly, one participant indicated that she differentiated whom she could confide in:

*“… but worried [it is] not confidential. I can trust doctors- they know better. But counselling- not really.” RY 105*

Some refugee youth also indicated that they did not think many of the services were culturally appropriate or likely to offer them the same services that were offered to Australian born youth:

*“…even with counselling or any of those kind of health services, they are using Western point of view and that is different to what other cultures believe so, it’s totally different” RY115*

*“If you are born in Australia you get more respect- they care more about you because you are part of them, one of them. Rather than coming from overseas you get treated... [trails off]…I think they care more about you if you are citizen and you are born in Australia.” RY110*

Not surprisingly, most participants (SP and RY) reported that language barriers were a significant obstacle to accessing services. Service providers said that some services did not have funding to use interpreters and those that did were sometimes encouraged to avoid using them wherever possible due to the high costs. Although this was a reported to be a barrier, service providers reported that young people were less likely to need interpreting services as many refugee youth acquired English skills faster than their older relatives. However, this was not always the case and one refugee youth participant reported his frustrations with not being able to communicate his experiences adequately:

“*They haven’t seen that stuff, so it’s hard to explain to them also. Some people who can’t speak English so they don’t know how to tell them, they don’t know how to say some words in English.” RY105*

There was some disparity between what interview and survey participants reported in relation to access to accredited interpreting services as the majority of survey participants reported that their clients do have access to interpreters (92.9%, *n =* 52).

Service providers also stated that it was common for refugee youth experiencing comorbidity to only access support services once they had reached a point of crisis and had been referred through emergency departments, crisis intervention services, homelessness agencies or the criminal justice system. Service providers generally agreed that intervening early was challenging because refugee youth did not usually access services during periods of stability:

*“Most people from these communities don’t seek help until they are dying. If it’s not serious, they don’t seek help until it is a crisis” SP 206*

Again, related to the fragmentation of services, it was reported that when a refugee young person did access a service, being referred back and forth between services was a common occurrence. Service provider participants stated that they witnessed an ongoing referral process where each service would determine that they were not suitable to deal with this client group or their presenting problem and would therefore refer them to other services. This was referred to as “handballing” and resulted in further disengagement of refugee background complex clients:

*“The biggest difficulty was trying to get mental health services on board. Often a response was, we won’t take that client on until you have dealt with their drug and alcohol issue…There are a lot of services out there that are happy to handball to the other service sector. They put it in the too hard basket.” SP 208*

*“that problem [back and forth referral] is even worse for the CALD communities, because apart from the fact that the services don’t necessarily have that cultural understanding, which makes it worse -but even within mainstream services, you’ve got mental health and drug and alcohol services trying to work together…” SP 205*

Some service providers recognised that the constant “handballing” of clients was not in the best interests of the young refugees with comorbidity:

*“…the idea that you would send somebody away to deal with a substance use issue and then deal with the mental health issue doesn’t work. You actually have to deal with them concurrently.” SP 202*

Consistent with the qualitative findings, the majority (67.5%, *n =* 27) of survey participants reported that young refugee background clients and potential clients face barriers to accessing their service. Similarly, most (77.5%, *n =* 31) considered that young people from refugee backgrounds do not have the same access to services as other young clients. Survey participants (*n =* 40) reported the top five barriers to accessing their service to be (1 = most significant barrier): 1. language, 2. shame and stigma, 3. unaware of service, 4. fear of deportation, and 5. fear of authority.

### Theme 3: Treatment and service delivery

Although type of therapeutic approach was not specifically explored, some service provider participants reported that Western therapeutic approaches may not always be appropriate for refugee background clients. However, others stated that it was possible to work therapeutically within Western modalities if the treatment was delivered in a flexible and culturally appropriate way.

The following subthemes relate to some of the organisational processes or systems in place reported to impede service access and effective service delivery. ‘Flexible service delivery’, meaning being flexible with inclusion criteria, rules around missed appointments or late arrivals, as well as where the service was delivered, was reported to be a facilitator to more effective engagement with this population.

A common concern reported by both service providers and refugee youth was that often the policies and procedures of services prevent refugee youth clients engaging in services. This related to both the presence of comorbidity and therefore not meeting inclusion criteria, appointment based services versus drop-in services, and time limitations such as a limited number of sessions and therefore not being able to accommodate CALD clients who may require more time to engage:

*“The organisation says ok this particular client seems like they are not interested in engaging with us, they look like they don’t need help but of course deep down they do need help, they just don’t know how to express it in a timely manner- in our time frame” SP207*

Other barriers concerning service delivery related to where the treatment was delivered and both service providers and refugee youth reported that offering the option of appointments outside of the office environment could facilitate access and engagement:

*“Sometimes the actual policies and procedures make it a barrier to these people accessing the services. Like the rigid “you’ve got this number of appointments, you can’t do this, your contract says you’re not allowed to work past 6pm or no home visits, no you can’t go to their house, they have to come here- well maybe they don’t feel safe coming here, maybe they would like me going out to meet in their environment” SP 205*

It was also reported that although some services were able to offer home visits, many were not which sometimes resulted in workers not adhering to policies and procedures in order to engage the client. This was not surprising given that other service providers spoke about the need for such flexibility:

*“I rarely see people in the office. Home visits, schools wherever they want to meet. I know that not all services are that flexible.” SP 202*

There was general agreement among refugee youth participants that offering flexibility in appointment location encouraged engagement and could even facilitate deeper communication. It was also suggested that changing the format from sitting down, face-to-face to walking side-by-side could enhance communication:

*“Maybe they meet somewhere else like in a park one day and not in the hospital every time. When you walk the environment it feels good and then you feel you can talk about whatever you want.” RY 106*

Although home visits were not common practice among the 15 service providers interviewed, the majority of survey participants reported their service allows home visits (71.4%, *n =* 40). In light of the qualitative data, it was encouraging that service managers reported they were able to offer a flexible service to their clients to facilitate greater access and engagement.

A common theme from the interviews with service providers and refugee youth concerned holistic care and consideration of clients’ non-clinical needs in addition to their MH and AOD issues. When services were able to offer this, it was reported to encourage engagement, continuity of care, and foster trust and the therapeutic relationship. However, funding barriers and limited resources were reported to impede this option and therefore result in discontinuity and reduced ability to engage in treatment. Holistic care was seen as an alternative and a solution to the problems associated with fragmented care:

*“I think that sometimes when services are funded so specifically “well no, we are not funded, it is not in our service agreement to work with a client experiencing mental health issues, it clearly states that all we work with is the drug and alcohol” and then you have the mental health services that say “no, no, we are not going to address the drug and alcohol issue, we are not going to address homelessness, we are only funded to help with mental health”... It’s really hard for the young person to understand why their issues have to be broken down the way they are, why they have to see so many services…. we have to look at problems holistically. Even though we are funded to work with … (omitted for confidentiality) we would still address their homelessness situation” SP 201*

Service providers often reported that without the option of offering holistic comorbidity services which have the capacity to address other needs, one way of reducing the disengagement resulting from siloed and fragmented services was to employ liaison, bi-cultural or CALD consultation workers to act as a buffer between all the relevant services:

*“Having some kind of overarching case manager or liaison officer who can work with that client side by side in referring them to different services, explaining the purpose, escorting them to their first appointments…We have a number of CALD liaison officers whose primary role is to just do that, start people off on their journey and guide them through” SP 201*

Approximately half (47.5%, *n =* 19) of the services reported that their service employed individuals in roles dedicated to working with CALD clients such as a CALD worker, cultural liaison, or CALD consultation worker. Although their role descriptions varied slightly, the primary goals for these positions were to act as a support and advocate for CALD clients and a cultural resource to other staff.

There were divergent perspectives between the refugee youth and service providers concerning possible involvement of the family in the treatment of the young people. Refugee youth participants described involving family members as highly undesirable and spoke of the fear young people experience when they think clinicians are going to involve the family. This was also reported to be a reason why young people did not seek help. Service provider participants, on the other hand, believed that where it was appropriate, possible and able to be negotiated, treatment involving the family was often more beneficial:

*“The more you involve the family the more it becomes successful.” SP 206*

Consistent with the interview findings, the majority of survey participants (67.5%, *n =* 27) believed that young people from refugee backgrounds with comorbidity do not get the same level of treatment as those who experience only a MH problem or an AOD problem. Further, only 30% (*n =* 12) of participants believed that refugee youth with comorbidity receive the same level of treatment as young people in the general population with comorbidity.

Survey participants (*n =* 40) also reported that the main difficulties of working with refugee youth clients are (top 5 in order, 1 = most significant difficulty): 1. managing language differences, 2. having access to sufficient bi-lingual resources, 3. negotiating family attitudes and perception of treatment, 4. managing cultural differences, and 5. negotiating clients attitudes and perception of treatment. These barriers are consistent with those reported by service providers during the interviews.

### Theme 4: Training and resources

Service providers reported a widespread unmet need for training in working with refugee background clients. Overwhelmingly, they described the lack of training offered by training institutions such as universities and vocational training establishments, as well as by the organisations for which they worked:

*“One of the biggest challenges I see is workers with limited cultural awareness, cultural competence in mainstream services and not necessarily through any fault of their own, just not understanding the challenges, the differences and even presentations of whether it is psychosis or other mental health issues. Even having experienced clinicians and doctors not understanding that that presentation may not be schizoaffective disorder, it might just be a reaction to torture and trauma, or to someone in their homeland who has just passed away…” SP205*

Refugee youth agreed with service providers saying that they thought it was necessary for workers to be trained in how to work effectively with refugee background individuals:

*“I would say to people, like a counsellor or a psychologist, to try to understand different cultures because you never know who you could be working with, so while they are doing their training and education… I’m sure they might do it but it’s still from a Western point of view and you really inhibit people from just accessing those kind of services and even if they do, they don’t feel satisfied” RY115*

*“You should learn about our country. You should use an interpreter. You should ask if they have any problems like coming to Australia, if they feel free or is something missing?” RY105*

It is important to note that service providers reported that even when CALD or refugee training was offered, it was usually optional. Service providers emphasised the need for widespread training:

*“Generally training in the use of interpreters is compulsory but generally the other stuff is not. So really we have to make it look as interesting as possible to sell it to everyone.” SP202*

Overall, the survey findings corroborated the interview findings regarding staff training for working with refugee background clients. Only 25% (*n =* 10) of managers reported that their staff received this type of training. More participants reported that their staff received training for working with CALD clients generally (65%, *n =* 26), however, service provider interview participants often commented that this was primarily focussed on working with Indigenous Australian clients. According to the survey data, the perceived competence of staff was low, with only 15% (*n =* 6) of survey participants reporting that staff within their service were adequately trained to provide treatment for refugee background clients and 40% (*n =* 16) reporting that there is room for improvement.

The discussions during the interviews with service providers about the need for training and upskilling the workforce were primarily centred on the need for training for working with refugee background clients rather than training for working with individuals with comorbidity. However, survey findings suggested that clinicians may not be adhering to best practice guidelines regarding detection of comorbidity and may not be adequately assessing refugee youth clients for comorbidity. Less than half of survey participants (48.7%, *n =* 19) reported that they screen all refugee youth clients to detect the co-occurrence of MH and AOD disorders, 20.5% (*n =* 8) reported they screen most clients, 10.3% (*n =* 4) reported screening some and 20.5% (*n =* 8) reported they do not screen any clients.

Service providers highlighted that often assumptions are made about a client’s cultural background, religion or traditional values, which leads the provider to believe there is no need to inquire about substance use:

*“That is part of the mindset as well, ‘they are Muslim so they don’t drink’. They are Islamic so they don’t have drug and alcohol problems, and that is part of Western mainstream thinking.” SP205*

### Government versus non-government

Interview participants suggested there were marked differences between the response of GO and NGO organisations to refugee youth with comorbidity, and this was confirmed by statistical tests which showed significant differences between GO and NGO services (Table 3).

**Table 3:** Comparison of Government and non-Government services

	Government		Non-Government		Total	Χ(df), *p* value	Effect size
	Yes *n* (%)	No or otherwise specified *n* (%)	Yes *n* (%)	No or otherwise specified *n* (%)	*n* (%)	Chi-Square (df), significance	(Cramer’s *V*)
Does your service allow home visits?	21 (37.5%)	13 (23.2%)	19 (33.9%)	3 (5.4%)	56 (100%)	3.960 (1), *p* = .047*	.266
Do your clients have access to accredited interpreters?	33 (58.9%)	1 (1.78%) (no/unsure)	19 (33.9%)	3 (5.4%)(no/ unsure)	56 (100%)	2.304^a^ (1), *p* = .129 Fisher’s exact test (2-tailed): *p* = .289	.203
Is your service adequately funded to provide treatment to refugee background clients?	8 (14.3%)	26 (46.4%)	9 (16.1%)	13 (23.2%)	56 (100%)	1.908 (1), *p* = .167	.185
Is your service adequately resourced to provide treatment to refugee background clients?	10 (17.9%)	24 (42.9%)	9 (16.1%)	13 (23.2%)	56 (100%)	.788 (1), *p* = .375	.119
Does your organisation collect data regarding if client is of refugee background?	5 (12.5%)	19 (47.5%)(no/ only country of birth)	8 (20%)	8 (20%) (no/ only country of birth)	40 (100%)	3.723 (1), *p* = .054	.305
Do your staff receive any training for working with CALD clients?	11 (27.5%)	13 (32.5%)	15 (37.5%)	1 (2.5%)	40 (100%)	9.689 (1), *p* = .002**	.492
Do your staff receive any training for working with refugee background clients?	3 (7.5%)	21 (52.5%)	7 (17.5%)	9 (22.5%)	40 (100%)	5^a^ (1), *p* = .025* Fisher’s exact (2-tailed) = .059	.354
Does your service employ 1 or more CALD/ cultural liaison/consultation or bi-cultural workers designated to work with CALD clients?	5 (12.5%)	19 (47.5%)	14 (35%)	2 (5%)	40 (100%)	17.109 (1), *p* = .000**	.654
In your opinion are the staff in your service adequately trained to provide treatment for refugee background clients?	0 (0%)	24 (60%) (No/There is room for improvement)	6 (15%)	10 (25%) (No/There is room for improvement)	40 (100%)	10.588^a^ (1), *p* = .001** Fisher’s exact test (2-tailed): *p* = .002**	.514
Do refugee clients and potential clients experience any barriers to accessing your treatment service?	17 (42.5%)	7 (17.5%)	10 (25%)	6 (15%)	40 (100%)	.304 (1), *p* = .581	.087

Fisher’s exact test is only calculated where 1 or more cells have a count less than 5

*p < .05 **p < .01

^a^1 or more cells contain a value less than 5 breaching the assumption of Chi-square test of Independence

## Discussion

This study sought to determine whether services in a particular region of South Australia were equipped to respond to refugee background youth with comorbidity, and to identify some of the barriers and facilitators to culturally responsive comorbidity care. It was apparent that whilst a number of services were attempting to be culturally responsive and meet the needs of refugee clients, there were significant gaps in the service response to young refugee background clients with comorbid MH and AOD problems as well as those with one condition. Some of the barriers reported in this research were consistent with recent literature outlining barriers to service provision for resettled refugee youth in Australia [47].

The gaps in service provision warrant immediate attention. As our key focus was on the provision of services to those experiencing comorbidity, the following discussion and identified solutions are central to that objective. When the themes and subthemes from the qualitative component were considered and integrated with the survey findings, three key areas were emphasised; organisational changes, policies and procedures; accessibility, engagement and treatment delivery; and workforce development. We discuss the integrated findings under these key domains.

### Organisational changes, policies and procedures

The National Practice Standards for the Mental Health Workforce (2013)for Australian nurses, psychologists, psychiatrists, social workers, and occupational therapists [48] emphasise cultural responsiveness and state that workers should use culturally appropriate assessment tools and demonstrate an awareness of the cultural issues which may impact upon assessment, care and treatment. Our findings suggest that these standards are not met by health professionals as there is a lack of awareness and confidence in the workforce about how to approach working with this population.

#### Fragmented services

The emphasis on the fragmentation of services by participants in the present study was not surprising given the existing literature highlighting that this is a widespread problem resulting from separate funding and organisation of MH and AOD services [49]. Our findings have drawn attention to an additional fragmentation of specialist (migrant/refugee) and mainstream services which creates the additional ‘run-around’ for comorbidity clients from refugee backgrounds. This is a population at great risk of ‘falling through the gaps’. The reported ‘handballing of complex clients’ given their refugee background and comorbidity status emphasises the need for policies and procedures to be produced for organisations and clinicians to be aware of this vulnerable group, develop sufficient competency in managing their difficulties and provide coordinated care.

The lack of service-level data collection concerning refugee background clients further exacerbates these problems. Without data it is difficult to determine needs, plan services, and justify additional funding, staff training and resources. Therefore, a paradoxical situation is evident. Unless organisations experience an increase in the number of young refugees accessing services, there is no argument to increase staff training, funding or access to necessary resources or to work on establishing connections with communities and promoting their service. However, without such funding and resources, there will continue to be significant access barriers in place, clinicians will continue to lack effective engagement strategies with this population, and the necessary community education, capacity building and service promotion will not occur.

#### Community engagement and interagency collaboration

As suggested by participants in this research, MH and AOD services need to take responsibility for community engagement and service promotion, and developing the necessary partnerships to facilitate this process. Participants from all data sources identified the need for mainstream services to collaborate with specialist agencies that have existing knowledge and links with CALD communities. A recent study in Sweden found that lack of collaboration between services was a major barrier to working effectively with refugee clients [50]. Creating formal or informal partnerships has been identified as a means for improving the provision of services to young people with comorbidity [25]. This need may be even greater in addressing service provision for refugee background young people with comorbidity. Participants who were successfully collaborating and communicating with other services or professionals reported smoother transitions between services for clients, increased accessibility, and greater continuity of care. Although the nature of competitive tendering for funding and grants was seen to hinder communication between services and workers and encourage the siloing of services, participants reported that partnerships and collaborative work could help overcome funding barriers and enable greater scope in community engagement initiatives. Young people also expressed the need for community development and capacity building work in order to increase awareness of problems and support services, reduce the fear and stigma around accessing them and provide them with a feasible way to make contact. A recent study of drug use among African youth in Victoria recommended targeted programs to improve health literacy to prevent drug use (specifically injecting drugs), increase awareness of MH problems, and reduce stigma among African youth [18]. Other researchers recommend creating strong partnerships between MH services, refugee communities, and social and settlement services [28, 51] and suggest using these partnerships to better coordinate interagency service planning and delivery for CALD clients with comorbidity [26].

#### Bi-cultural workers and culture brokers

Our findings highlight that where possible, services would benefit from hiring CALD or bi-cultural workers to act as advisors, culture brokers and a resource for staff and to improve interagency liaison and collaboration. Survey data suggested this had already been initiated in some services and interview participants spoke of the resulting benefits. Our survey analyses suggest that this occurs more in NGOs than GOs. Increasing the number of bilingual health professionals in services has previously been recommended to improve comorbidity care for CALD clients [26]. CALD workers can document, interpret and provide valuable insights into hidden, nuanced and sensitive material, elements of distress and deterioration that are unlikely to be detected by mainstream workers. Kirmayer et al. [51] describes a stepped process of working effectively with interpreters and culture brokers and outlines how this can improve communication and reduce some of the commonly reported language barriers. The National Practice Standards specifically state that MH workers should liaise or work collaboratively with CALD ‘care partners’ such as religious and spiritual leaders, traditional healers and community-based organisations, and bilingual counsellors [48]. Participants commonly suggested hiring liaison officers as a solution to the difficulties clients experience navigating multiple services. Further, many participants suggested integrating the roles of CALD workers and liaison workers to prevent disengagement by refugee clients when they are engaged with multiple services. Some services reported already trialling this, the outcome of which should be evaluated by future research. The Mental Health Service Guidelines states that the MH services must deliver services that take into account the cultural and social diversity of its consumers and meet their needs [52]. Given that the data highlights that this objective is not being sufficiently met, evaluations and changes at an organisational level are recommended.

### Accessibility, engagement and treatment delivery solutions

The study findings highlighted the small numbers of refugee youth with comorbidity attending relevant services. Given that this did not reflect the representation of refugee youth in the northern suburbs, it is a significant concern. Across our survey and interview findings, a number of barriers to access were reported with emphasis placed on resources, funding, screening for comorbidity, cultural responsiveness, fear, shame and stigma, and awareness of services.

Interviews with young people indicated that they were either not aware of support services or if they were, many reported a lack of faith in them for a variety of reasons. Some refugee young people interviewed stated that they would rather speak with friends about MH difficulties or AOD use, and that they would not trust services because they feared family involvement.

Young people also reported that MH was not a priority. If the clinicians were not able to simultaneously assist with migration issues, housing issues, social, educational and occupational issues, then they quickly disengaged. A recent qualitative study interviewing experienced therapists working with refugee clients found that meeting the practical resettlement needs of clients was vital in acknowledging the wider socio-political context relevant to the individual [53]. The National Comorbidity Clinical Guidelines also argue that clients with complex needs such as comorbidity require a holistic approach to treatment and that clinicians should assist with other needs where possible [4]. We have previously emphasised the importance of a holistic approach to this particular client group [54]. Flexibility with regards to appointment location was identified by participants as a critical aspect of engagement and also has the potential to reduce the perception of stigma for the young person requiring the service. This finding is consistent with recent research that concluded services should have the “flexibility and accessibility to engage the child, and mental health input should always be integrated with welfare, education and physical health services” [55].

#### Professionals’ knowledge and explanatory models

There was agreement among all participants that counsellors and psychologists needed to understand where their clients have come from and show an interest in their culture and refugee past. We conceptualise this knowledge as a combination of trauma-informed and culture-informed care. It requires professionals to explore the meanings that clients attach to their experiences and problems, have an understanding and an interest in their clients’ past and cultural background, and an appreciation of the refugee experience, which includes the ongoing impact of the journey, and the ongoing difficulty of adjusting to a new culture.

Our findings indicate that there is still a need for MH and AOD professionals to understand the explanatory models of CALD individuals. Kleinman [56] defines explanatory models as understandings or explanations of illness or treatment within the context of social and cultural beliefs and history. This requires an understanding of the way in which symptoms are presented, when, how and why help is sought, and what is considered a good outcome. The Diagnostic Manual for Mental Disorders Fifth Edition (DSM 5) includes a section on cultural formulation and offers a series of questions (the cultural formulation interview) which enables clinicians to obtain information about the impact of culture and emphasises explanatory models in various domains [57]. Research has found that consideration of the cultural formulation in assessment is useful in improving diagnostic accuracy [58]. These approaches to assessment and formulation could be easily incorporated into future training for AOD and MH service providers.

### Workforce development

The lack of appropriate training was evident from both the survey responses and the interviews. The need for workforce development was apparent in two particular areas; training on working with individuals from refugee backgrounds, and training on the assessment, diagnosis and best treatment for individuals experiencing comorbidity. We suggest that the integration of these two areas of training may serve to improve the provision of services to this population.

#### Training regarding working with refugee clients

There is a clear need for health professionals to be more aware of the factors that impede access to services, effective engagement, and continuity of care for clients both with comorbidity and from refugee backgrounds. Many organisations have compulsory training for working with indigenous clients only, and service providers commented that given the demographics of the region, their skill base does not accurately reflect the diversity of the local population. The majority of managers reported that their staff did not receive opportunities to gain knowledge and skills in relation to this population and further, did not perceive their staff to be adequately trained to work with refugee background clients, so up-skilling the workers to reflect the large CALD and refugee background population should be considered a priority. This may be more pertinent to GOs given that our survey found that none of the managers in GOs perceived their staff to be adequately trained to work with refugee youth. Bӓӓrnhielm et al. [50] argued that access to care by refugees is influenced by professionals’ knowledge about cultural aspects of patients’ expressions and understanding of mental distress. We would argue this relates to the need for health professionals to understand differing explanatory models. Bӓӓrnhielm [50] evaluated the impact of cross cultural training on working with refugees and found an increased ability to understand the vulnerability and contextualised health of newly arrived refugees, as well as increased empathy with ways of expressing distress which were unfamiliar to them. However, they acknowledged that this training was insufficient because it had no impact on the lack of collaboration between services and workers, the latter of which was seen as a more significant barrier. The authors also reported that participants perceived the lack of collaboration to be an organisational responsibility. Similarly, Colucci et al. [47] stated that “enhancing the cultural competence of services is important but not sufficient to ensure children and young people in need are able and willing to access assistance” (p.17). Although there is insufficient evidence of the benefits of improving the cultural competency of services in improving outcomes [59], our findings suggest that the self-perceived lack of competency by staff is leading to the “handballing” of clients, resulting in further disengagement and loss of hope by youth, signifying that training is certainly necessary.

#### Training regarding Comorbidity

Almost half of the survey participants reported screening their refugee youth clients for comorbidity. This finding was promising, however, given the prevalence of comorbidity in Australia and mental health services, it would be ideal and considered best practice if clinicians screened all of their clients. Findings indicated that there was an assumption by many workers that if a client was Muslim or from a country with conservative views of AODs that they would assume there was no AOD use. There is a need for clinicians to be aware of the high prevalence of comorbidity in order to identify if AOD use is contributing to or maintaining the problems, and if there is a need to involve other workers and services. Given the high prevalence of comorbidity in service settings, many guidelines have recommended compulsory training for clinicians in order to be equipped with skills in screening for and treating comorbidity [4, 9].

### Divergent findings

Overall, there was great convergence between the three methods of generating data. However, through the comparison of findings from different data sources, there were some inconsistencies between what was reported by survey participants and what was reported by service providers and youth in the qualitative interviews. For example, although the majority of service managers reported they had access to interpreters, service providers in the interviews stated they are often encouraged to avoid using them due to the associated high costs. Refugee youth participants reported that they often act as the interpreter for relatives and friends accessing health services, suggesting they may be filling the interpreter role. Such divergent findings support the need for further research.

### Limitations

Although yielding important findings and adding to the paucity of literature on this topic, this study was not without limitations. Interview participants were recruited using purposive and snowball sampling methods which are common sampling methods used in qualitative research. This limits the generalisability of the results. However, given the research aims were to explore participants’ experiences and opinions, this method was appropriate. Further, the survey response was overwhelmingly from the MH sector, or from services that identified as being ‘combined’. Therefore the findings might be limited to only MH or combined services. However, it should be noted that there certainly are fewer AOD services and programs and therefore the low response rate from AOD services may reflect the fact there are less AOD services and service providers. Additionally, we also acknowledge the potential for selection bias because it is possible that only those with a particular interest in the research topic participated in the survey (much like the trend for seeking professional development in this area). Although we made concerted efforts to contact all eligible services in the region, we cannot be confident this was a broad sample of the services. Further, we were unable to determine if multiple people from the same organisation were responding to the anonymous survey as many services had various management positions.

The relatively small number of individuals who participated in the interviews and online survey, and the fact that the study was limited to a particular region of SA, does not allow us to make broader generalisations. It is not uncommon for refugees to be resettled in areas of social disadvantage, often on the outskirts of cities where there is affordable housing. Therefore the findings from this research may well apply to other areas across Australia with similar demographics, particularly if services are structured similarly and there are similar funding limitations and limited training opportunities.

## Conclusion

Considering the large number of refugee background youth who reside in northern Adelaide, it is essential that MH and AOD services have the capacity to respond appropriately to the needs of this diverse community. Our research has found there are significant gaps in the service response to this population and findings highlight a general and widespread lack of cultural responsiveness by services in dealing with refugee youth clients experiencing comorbidity. The implications of these findings have been discussed and we have reported various solutions which warrant consideration by Governments, organisations, and MH and AOD staff.

## Abbreviations

AOD: Alcohol and other drug
CALD: Culturally and linguistically diverse
GO: Government Organisation
MH: Mental health
NGO: Non-Government Organisation
